# LFA-1 Mediates Cytotoxicity and Tissue Migration of Specific CD8^+^ T Cells after Heterologous Prime-Boost Vaccination against *Trypanosoma cruzi* Infection

**DOI:** 10.3389/fimmu.2017.01291

**Published:** 2017-10-13

**Authors:** Camila Pontes Ferreira, Leonardo Moro Cariste, Fernando Dos Santos Virgílio, Barbara Ferri Moraschi, Caroline Brandão Monteiro, Alexandre M. Vieira Machado, Ricardo Tostes Gazzinelli, Oscar Bruna-Romero, Pedro Luiz Menin Ruiz, Daniel Araki Ribeiro, Joseli Lannes-Vieira, Marcela de Freitas Lopes, Mauricio Martins Rodrigues, José Ronnie Carvalho de Vasconcelos

**Affiliations:** ^1^Molecular Immunology Laboratory, Center of Molecular and Cellular Therapy, São Paulo, Brazil; ^2^Department of Microbiology, Immunology and Parasitology, Federal University of São Paulo (UNIFESP), São Paulo, Brazil; ^3^Department of Biosciences, Federal University of São Paulo, São Paulo, Brazil; ^4^René Rachou Research Center, Fiocruz, Minas Gerais, Brazil; ^5^Division of Infectious Disease and Immunology, Department of Medicine, University of Massachusetts Medical School, Worcester, MA, United States; ^6^Department of Microbiology, Immunology and Parasitology, Federal University of Santa Catarina, Florianópolis, Brazil; ^7^Biology Interactions Laboratory, Oswaldo Cruz Institute, Fiocruz, Rio de Janeiro, Brazil; ^8^Institute of Biophysics Carlos Chagas Filho, Federal University of Rio de Janeiro, Rio de Janeiro, Brazil

**Keywords:** vaccination, *Trypanosoma cruzi*, migration, integrins, specific CD8^+^ T cells

## Abstract

Integrins mediate the lymphocyte migration into an infected tissue, and these cells are essential for controlling the multiplication of many intracellular parasites such as *Trypanosoma cruzi*, the causative agent of Chagas disease. Here, we explore LFA-1 and VLA-4 roles in the migration of specific CD8^+^ T cells generated by heterologous prime-boost immunization during experimental infection with *T. cruzi*. To this end, vaccinated mice were treated with monoclonal anti-LFA-1 and/or anti-VLA-4 to block these molecules. After anti-LFA-1, but not anti-VLA-4 treatment, all vaccinated mice displayed increased blood and tissue parasitemia, and quickly succumbed to infection. In addition, there was an accumulation of specific CD8^+^ T cells in the spleen and lymph nodes and a decrease in the number of those cells, especially in the heart, suggesting that LFA-1 is important for the output of specific CD8^+^ T cells from secondary lymphoid organs into infected organs such as the heart. The treatment did not alter CD8^+^ T cell effector functions such as the production of pro-inflammatory cytokines and granzyme B, and maintained the proliferative capacity after treatment. However, the specific CD8^+^ T cell direct cytotoxicity was impaired after LFA-1 blockade. Also, these cells expressed higher levels of Fas/CD95 on the surface, suggesting that they are susceptible to programmed cell death by the extrinsic pathway. We conclude that LFA-1 plays an important role in the migration of specific CD8^+^ T cells and in the direct cytotoxicity of these cells.

## Introduction

Chagas disease, caused by the intracellular parasite *Trypanosoma cruzi*, is a major public health problem, with about seven million people infected worldwide (1). CD8^+^ T cells are crucial for controlling the multiplication of intracellular pathogens such as *T. cruzi*. These cells control the infection by secreting cytokines such as IFN-γ and TNF-α, or by direct cytotoxicity against infected target cells (2). The heterologous prime-boost vaccination strategy has shown significant results in the induction of specific CD8^+^ T cells and the generation of an optimal protective immune response. Among several possible combinations of vectors for this type of immunization, we used a plasmid vector for priming and an adenovirus-Ad5 vector (replication-defective human Ad type 5) for boosting, both containing an insertion of the ASP-2 gene (*T. cruzi*’s amastigote surface protein 2 gene). This type of immunization was capable of protecting A/Sn mice that are highly susceptible to experimental infection with *T. cruzi* (3, 4).

The results obtained in preclinical experimental models with heterologous prime-boost immunization have boosted recent clinical trials (5–10). In 2013, the first results of a Phase II clinical trial were published. In that study, a number of volunteers, who were vaccinated with plasmid DNA followed by immunization with Ad5, both encoding the genes of the apical membrane antigen 1 and the immunodominant surface protein of the *Plasmodium falciparum* circumsporozoite protein, developed immunity to malaria (11).

To the CD8^+^ T cells exert their effector function, these cells must migrate to non-lymphoid peripheral tissues where the infection occurs. Our group recently demonstrated that the protection generated by heterologous prime-boost immunization regimen depends on the recirculation of specific CD8^+^ T cells, since immunized and protected A/Sn mice became susceptible to the experimental challenge with *T. cruzi* after FTY720 drug treatment (12). This immunosuppressive drug reduces lymphocyte recirculation by altering T cell signaling *via* sphingosine-1-phosphate receptor-1 (S1Pr1). This leads in sustained inhibition of S1Pr1 signaling, trapping T cells within the secondary lymphoid with no impairment of T cell activation (12, 13). Based on this knowledge, we hypothesized that other molecules, such as integrins, could be involved in the CD8^+^ T cell migration. The integrins are heterodimers that composed of an alpha and beta chain; LFA-1 is composed of αLβ2 (CD11a/CD18) chains, and VLA-4, of α4β1 (CD49d/CD29) chains. These molecules play an important role in the formation of immunological synapses and signal transduction, which result, for example, in cell migration, activation, and/or proliferation (14, 15). During transendothelial migration, chemokine-triggered activation of both LFA-1 and VLA-4 leads them to change their conformations and strongly bind to intercellular adhesion molecules (ICAMs and VCAMs, respectively) on endothelial cells and, thus, migrate into the tissues (16). In β2 integrin-deficient mice, LFA-1 shows a significant reduction in the *in vitro* lymphocyte migration, strengthening the role of this molecule in leukocyte migration (17). The LFA-1 role in lymphocyte migration has also been demonstrated in the experimental autoimmune encephalomyelitis, in which regulatory CD4^+^ T cells can migrate to the CNS *via* LFA-1 (18). Its role has also been demonstrated in allografts, and the antagonism of this molecule is a very effective inhibitor of acute rejection, thus prolonging allograft survival in rodents (19). VLA-4 has also been studied in liver allograft rejections, where it seems responsible for the migration of effector CD8^+^ T cells and transplant rejection along with LFA-1 (20, 21). During infection by intracellular parasites such as *T. cruzi*, specifically by the Colombian strain, there is a predominance of effector CD8^+^ T lymphocytes (CTLs) with high expression of LFA-1 and VLA-4 in the myocardium of infected mice (22). In addition, the high expression of LFA-1 on the surface of Pfn^+^CD8^+^ T cells during the acute and chronic phases has been demonstrated (23). However, the dominance of these cells in cardiac tissue favors the progression of the inflammatory reaction, culminating in Chronic Chagas heart disease (24).

Herein, we tested whether LFA-1 and VLA-4 integrins were key mediators for T cell-mediated protective immunity against *T. cruzi* infection. For that purpose, mice were vaccinated with heterologous prime-boost vaccine (recombinant plasmid DNA/AdHu5), challenged and treated with blocking antibodies to LFA-1 and/or VLA-4. Our results demonstrate that LFA-1, but not VLA-4, is essential for protective immune response of highly susceptible mice against *T. cruzi* infection. Also, the study demonstrated that LFA-1 mediates CD8^+^ T cells migration into infected tissues, such as the heart, and plays an important role in CD8^+^ T cells cytotoxicity for parasite clearance.

## Materials and Methods

### Ethics Statement

This study was carried out in strict accordance with the recommendations in the Guide for the Care and Use of Laboratory mice of the Brazilian National Council of Animal Experimentation (http://www.mctic.gov.br/mctic/opencms/textogeral/concea.html). The protocol was approved by the Ethical Committee for Animal Experimentation at the Federal University of Sao Paulo (Id # CEP 7559051115).

### Mice and Parasites

Female 5- to 8-week-old A/Sn or C57BL/6 mice were purchased from the Federal University of São Paulo. ICAM-1-deficient mice were kindly supplied by Dr. João Santana, Ribeirão Preto School of Medicine-FMPR. Parasites of the Y strain of *T. cruzi* were used in this study (2, 3). Blood trypomastigotes of the Y strain of *T. cruzi* were maintained by weekly passages in A/Sn mice at the Xenodiagnosis Laboratory of Dante Pazzenese Cardiology Institute. Bloodstream trypomastigotes were obtained from mice infected 7–28 days earlier with parasites of the Y strain. For *in vivo* experiments, each mouse was inoculated with 150 trypomastigotes (A/Sn) or 10^4^ trypomastigotes (C57BL/6) diluted in 0.2 mL phosphate-buffered saline (PBS) and administrated subcutaneously (s.c.) in the base of the tail. Parasitemia was determined by collecting 5 μL of blood, and parasites were counted on the light microscope (25).

### Immunization Protocol

In this study, we used the heterologous prime-boost immunization protocol with plasmid pIgSPCl.9 and the human replication-defective adenovirus type 5 containing the ASP-2 gene, as described previously (3, 26). Briefly, this immunization consists of a dose of plasmid DNA as a prime (pcDNA3 control or pIgSPClone9). The mice were intramuscularly inoculated (i.m.) with 50 µg of plasmid DNA into each *tibialis anterioris* muscle. Three weeks after the first immunization, mice were boosted with 2 × 10^8^ plaque-forming units of the adenoviral vectors Adβ-gal or AdASP-2. Both injections were performed *via* intramuscular route (tibialis anterior muscle).

### Peptide

TEWETGQI peptide was synthesized by GenScript and obtained at purity higher than 95%. The TEWETGQI epitope expressed on ASP-2 surface is target of CD8^+^ T cells and was identified previously (27). It was used for specific CD8^+^ T cell stimulation *in vitro* and *ex vivo*. The H2K^K^-TEWETGQI multimer, labeled with fluorophore APC, was purchased from Immudex (Copenhagen, Denmark) and used for specific CD8^+^ T cell detection in tissues.

### Treatment with Monoclonal Antibodies

Anti-LFA-1 (anti-CD11a, clone M17-4) and anti-VLA-4 (anti-CD49d, clone PS/2) monoclonal antibodies were purchased from BioXcell; in addition, we used Rat IgG2a (clone 2A3) isotype control. The *in vivo* treatment was performed with 10 i.p. injections of 250 µg of mAb/mouse (every 48 h after infection, until day 20 after infection). The concentration of LFA-1 used for *in vivo* treatment was the same used by Reisman et al. (28). To evaluate the efficiency of LFA-1 integrin blockade, C57BL/6 mice were infected with 10^4^ trypomastigote forms of Y strain, and 12 days post infection, the splenocytes were harvested and incubated *in vitro* for 24 h at 30°C with monoclonal 250 µg/mL of 2A3 isotype control or anti-LFA-1 in complete medium [1% NEAA, 1% l-glutamine, 1% vitamins and 1% pyruvate, 0.1% 2-ME, 10% fetal bovine serum (FBS) (HyClone)]. After incubation, splenocytes were washed and labeled with anti-CD8 PerCP (clone 53-6.7, BD) and anti-CD11a FITC (clone 2D7, BD), fixed with 1% paraformaldehyde and analyzed by flow cytometry. Concomitantly, we also evaluated the blockade of the LFA-1 molecule stimulating splenocytes *in vitro* with 1 µg/mL anti-CD3 (clone 145-2C11, eBioscience) in complete medium for 72 h at 37°C and 5% CO_2_. On the second day of incubation, 250 µg of 2A3 isotype control or anti-LFA-1 monoclonal antibodies were added to the culture. On the third day of culture, cells were harvested and labeled with anti-CD8 PerCP and anti-CD11a FITC for flow cytometric analysis. LFA-1 expression was performed on gated CD8^+^ T cells, according to Figures S1A,B in Supplementary Material, treatment with monoclonal anti-LFA-1 blocked most LFA-1 molecule expressed on activated CD8^+^ T cells and after anti-LFA-1 FITC staining there was a lower CD11a MFI on the surface of these cells, indicating that there is competition between anti-LFA-1 monoclonal antibodies used for *in vivo* blocking (clone M17-4) and anti-CD11a FITC (clone 2D7, BD) used for flow cytometry labeling.

### Real-time PCR

Hearts, livers, and spleens from the *T. cruzi*-infected, immunized, and/or treated mice with anti-LFA-1 A/Sn were used for extracting DNA. The extraction protocol, the specific primers for a satellite DNA region of the parasite, and the RT-PCR reaction using the TaqMan Universal Master Mix II with UNG were adapted from Piron and colleagues (29). For the race plates, we used StepOnePlus (Applied Biosystems^®^), and distilled water for negative control reaction.

### Enzyme-Linked Immunospot (ELISpot) Assay

Sterile PBS containing 10 µg/mL of anti-mouse IFN-γ monoclonal antibody (clone R4-6A2, Pharmingen) was added onto nitrocellulose 96-well flat-bottom plates; after 24 h, the plates were washed with RPMI and blocked with RPMI containing 10% FBS for 2 h. Following, 1 × 10^6^ responder cells from spleen, liver, or lymph node were incubated with 3 × 10^5^ antigen-presenting cells in complete medium [1% NEAA, 1% l-glutamine, 1% vitamins and 1% pyruvate, 0.1% 2-ME, 10% FBS (HyClone), and 20 U/mL mouse recombinant IL-2 (SIGMA)]. The plate was incubated in the presence or absence of 10 µM of peptide TEWETGQI. After 24 h, the plates were washed three times with PBS, and five times with PBS–Tween 20 (0.05% Tween). Each well received biotinylated anti-mouse monoclonal antibody (clone XMG1.2, Pharmingen) diluted in PBS-0.05% Tween 20 at a final concentration of 2 µg/mL. The plates were incubated with streptavidin-peroxidase (BD) and developed by adding peroxidase substrate (50 mM Tris–HCl, pH 7.5, containing 1 mg/mL DAB and 1 µL/mL 30% hydrogen peroxide, both from SIGMA). The number of IFN-γ-producing cells was determined using a stereoscope.

### Intracellular Cytokine Staining

Two million cells from the spleen, lymph node, or liver were treated with ACK buffer (NH_4_Cl, 0.15 M; KHCO_3_, 10 mM; Na_2_-EDTA 0.1 mM; pH = 7.4). ICS was performed after *in vitro* culture of splenocytes in presence or absence of 10 µM of peptide TEWETGQI as described previously (25). Cells were washed three times in plain RPMI and resuspended in cell culture medium consisting of RPMI 1640 medium supplemented with 10 mM HEPES, 0.2% sodium bicarbonate, 59 mg/L of penicillin, 133 mg/L of streptomycin, 10% HyClone FBS, 2 mM l-glutamine, 1 mM sodium pyruvate, 55 µM 2-mercaptoethanol. The viability of the cells was evaluated using 0.2% trypan blue exclusion dye to discriminate between live and dead cells. Cell concentration was adjusted to 2 × 10^6^ cells/mL in cell culture medium containing CD107a FITC antibody (clone 1D4B, BD), anti-CD28 (clone 37.51, BD), BD Golgi-Plug (1 µL/mL), and monensin (5 µg/mL) and incubated no longer than 12 h in V-bottom 96-well plates (Corning) in a final volume of 200 µL in duplicate, at 37°C in a humid environment containing 5% CO_2_. After 12 h incubation, cells were stained for surface markers with anti-CD8 PERCP antibody (clone 53-6.7, BD) on ice for 30 min. To detect IFN-γ, TNF or granzyme B by intracellular staining, cells were then washed twice in buffer containing PBS, 0.5% bovine serum albumin (BSA), and 2 mM EDTA, fixed and permeabilized with BD perm/wash buffer. After being washed twice with BD perm/wash buffer, cells were stained for intracellular markers using APC-labeled anti-IFN-γ (clone XMG1.2, BD), PE-labeled anti-TNF-α (clone MP6-XT22, BD), and anti-granzyme B PE (clone GB11, INVITROGEN) for 20 min on ice. Finally, cells were washed twice with BD perm/wash buffer and fixed in 1% PBS–paraformaldehyde. At least 700,000 cells were acquired on a BD FACS Canto II flow cytometer and then analyzed with FlowJo. Figures S3A,B in Supplementary Material shows the representative ICS gate strategies.

### Purification of Liver and Heart Lymphocytes

The perfused liver was lysed with collagenase buffer composed of 0.2 mg/mL collagenase IV (SIGMA), 0.02 mg/mL DNase (SIGMA), and 5% FBS. The leukocytes were separated on a 35% Percoll gradient (GE Healthcare), followed by centrifugation at 600 × *g* for 20 min and at 4°C. The pellet was suspended in RPMI 1640 (SIGMA) with 10% FBS (30). For the purification of the lymphocytes of the heart, we followed the protocol of Gutierrez et al. (31). Briefly, hearts collected from five mice at day 20 d.p.i. were minced, pooled, and incubated for 1 h at 37°C with RPMI 1640, supplemented with NaHCO_3_, penicillin–streptomycin gentamicin, and 0.05 g/mL of liberase blendzyme CI (Roche, Basel, Switzerland). The organs were processed in a Medimachine (BD Biosciences) in PBS containing 0.01% BSA. After tissue digestion and washes, cell viability was assessed by trypan blue exclusion, counted in a hemocytometer.

### Flow Cytometry Analysis

Splenocytes were treated with ACK buffer for red cell lysis and washed with RPMI with 10% FBS. The spleen, heart, lymph node, and liver cells were stained with H2K^k^-TEWETGQI multimer for 10 min at RT. The cell surface was stained for 30 min at 4°C. The following antibodies were used for surface staining: anti-CD3 APCcy7 (clone 145-2C11, BD), anti-CD8 PERCP or anti-CD8 PACIFIC BLUE (clone 53-6.7, BD), anti-CD11a FITC (clone 2D7, BD), anti-CD11c APCcy7 (clone HL3, BD), anti-CD44 FITC (clone IM7, BD), anti-CD62L PE (clone MEL-14, BD), anti-CXCR3 PERCP/Cy5.5 (clone 173, BioLegend), anti-CD27 FITC (clone LG3A10, BD), anti-CD4 PEcy7 (clone RM4-5, BD), anti-KLRG1 FITC (clone 2F1, eBioscience), anti-CD49d PEcy7 (clone R1-2, BD), anti-CD69 PERCP (clone H1.2F3, BD), anti-CD43 PEcy7 (1B11, BioLegend), anti-CD95 PEcy7 (clone JO2, BD), anti-CD25 FITC (clone LG3A10, BD), anti-CD127 PE (clone SB/199, BD), anti-CD122 FITC (clone TM-β1, BD), anti-CD38 PE (clone 90, BD), anti-β7 PERCP (clone FIB27, BioLegend), anti-CD31 FITC (clone MEC 13.3, BD), anti-CD272 PE (clone 8F4, eBioscience), anti-PD-1 FITC (clone J43, eBioscience), anti-CTLA-4 PE (clone UC10-4B9, eBioscience), and anti-CCR7 PE (clone 4B12, BD). At least 500,000 cells were acquired on a BD FACS Canto II flow cytometer and analyzed with FlowJo 8.7.

### *In Vivo* Proliferation Assay

A/Sn were immunized with ASP-2 using the heterologous “prime-boost” vaccination regimen and infected with 150 trypomastigotes forms of *T. cruzi*. At the moment of infection, mice were treated with monoclonal antibodies (LFA-1 or 2A3 isotype control) and 2 mg of BrdU (5-bromo-2′-deoxyuridine, SIGMA) by route i.p., at every 48 h, until the 20th day after challenge. Then, 2 × 10^6^ splenocytes were treated with ACK buffer for red cell lysis, washed with RPMI plus 10% FBS, and stained with H2K^k^-TEWETGQI multimer and anti-CD8 antibody. The specific CD8^+^ T cells were stained according BrdU-FITC Kit protocol (BD Pharmingen) for analysis of BrdU incorporation. A minimum of 700,000 cells were acquired on a BD FACS Canto II flow cytometer and analyzed with FlowJo 8.7.

### *In Vivo* Cytotoxicity Assay

For the *in vivo* cytotoxicity assays, splenocytes collected from naive A/Sn mice were treated with ACK buffer to lyse the red blood cells, as described by Silverio et al. (23). The cells were divided into two populations and were labeled with the fluorogenic dye carboxyfluorescein diacetate succinimidyl diester (CFSE; Molecular Probes, Eugene, OR, USA) at a final concentration of 10 µM (CFSE^high^) or 1 µM (CFSE^low^). CFSE^high^ cells were coated with 2.5 µM of the TEWETGQI ASP-2 peptide for 40 min at 37°C. CFSE^low^ cells remained uncoated. Subsequently, CFSE^high^ cells were washed and mixed with equal numbers of CFSE^low^ cells before intravenous injection (2 × 10^7^ cells per mouse) into *T. cruzi*-infected, immunized and/or treated mice with anti-LFA-1 A/Sn recipients that were sedated with diazepam (20 mg/kg). Spleen cells from the recipient mice were collected at 20 h after adoptive cell transfer and fixed with 1.0% paraformaldehyde. At least 100,000 cells were acquired on a BD FACS Canto II flow cytometer and analyzed with FlowJo 8.7. The percentage of specific lysis was determined using the following formula:

$$\%\text{ lysis}=1-\frac{\left(\%{\text{CFSE}}^{\text{high}}\;{\text{infected/\%CFSE}}^{\text{low}}\text{ infected}\right)}{\left(\%{\text{CFSE}}^{\text{high}}\;{\text{naive/\%CFSE}}^{\text{low}}\text{ naive}\right)\times 100}.$$

### Histology and Immunohistochemistry

The mice’s heart, spleen, and liver were fixed in 10% formalin, and then dehydrated, embedded in paraffin blocks, and sectioned on a microtome. Staining was obtained with hematoxylin and eosin, and the number of amastigotes nests was quantified using a light microscope with 40× objective lens. Overall, 50 fields/group were counted. For immunohistochemistry the hearts of the animals were removed and frozen in Tissue-Tek O.C.T. (Sakura Finetek), and the 7 µm thickness cuts were made in the cryostat (Leica) and then fixed in ice-cold acetone for 15 min. The samples were stained with 20 µg of the biotinylated anti-CD8 antibody (clone 53-6.7, RD systems) for 12 h in the wet chamber, and after incubation was labeled with streptavidin Alexa Fluor^®^ 488 (Thermo Fischer) at the concentration of 0.5 mg/mL, diluted 1:100 for 1 h and room temperature. The DAPI (4′,6-diamidino-2-phenylindole, SIGMA) dye was used for labeling the 5 mg/mL cell nucleus, diluted 1:1,000 for 15 min at room temperature. The images were acquired in the Confocal Leica TCS SP8 CARS microscope of the Institute of Pharmacology and Molecular Biology (INFAR) of the Paulista School of Medicine of the Federal University of São Paulo. The images were obtained using the 63× objective and processed by the ImageJ program.

### Statistical Analysis

The number of parasites/mL corresponding to the peak of parasitemia, the number of IFN-γ-producing cells (ELISPOT), and the absolute number of CD8^+^ T cells were compared by analysis of unidirectional variance (ANOVA); subsequently, the Tukey’s HSD test was used. To compare the survival of mice after challenge with *T. cruzi*, we used the Log-rank test. The receptor expression was compared using MFI (mean fluorescence intensity), and the *naive* group MFI was taken as the baseline. MFI was determined by the FlowJo software. Differences were considered significant when *P* value was <0.05.

## Results

### LFA-1 Is Essential for Survival of A/Sn Mice during the Experimental Challenge with *T. cruzi*

**Figure 1:**
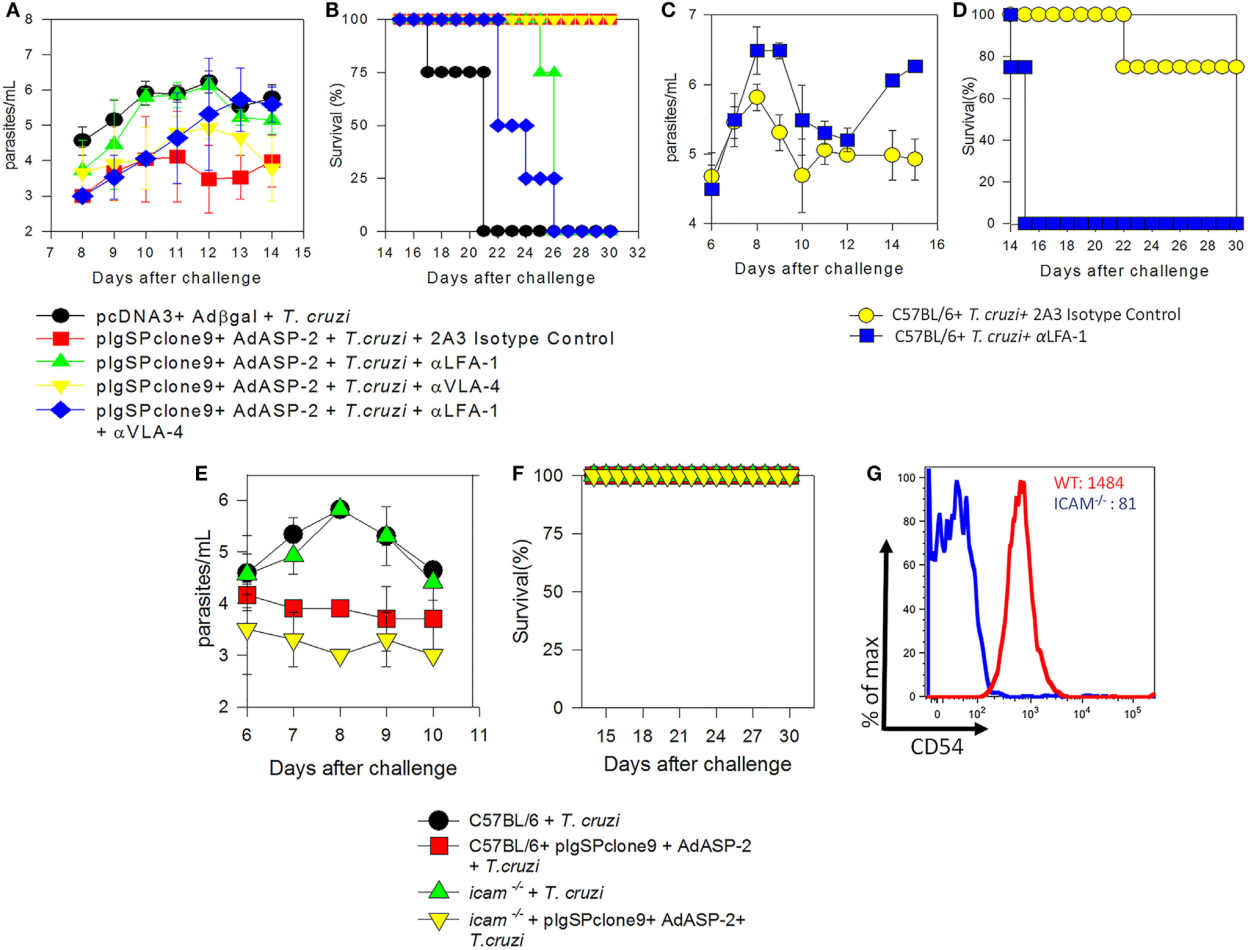
LFA-1 blockade increases blood parasitemia and culminates in the death of A/Sn and C57BL/6 mice. A/Sn-, C57BL/6-, and intercellular adhesion molecule (ICAM)-deficient mice were immunized with plasmids pcDNA3 and/or pIgSPclone9 and adenoviral vectors Adβgal and AdASP-2. Fifteen days after the last immunization dose, mice were infected with 150 (A/Sn) and/or 10^4^ (C57BL/6 and ICAM mice) blood trypomastigotes of the Y strain of *Trypanosoma cruzi*. Anti-VLA-4 and/or LFA-1 treatment was performed every 48 h after infection until the 20th days after infection. **(A)** Blood parasitemia was assessed, and values were log transformed and parasitemia data for each mice group are represented as mean ± SD for each group (*n* = 4). Values from peak parasitemia (day 12) were compared by one-way ANOVA and Tukey’s HSD tests, and the results were as follows: Significant differences were found between the red in green groups (*P* < 0.05). There were no significant differences between the blue group and the green group (*P* > 0.05). **(B)** Kaplan–Meier curves for survival of the different groups were compared using the log-rank test. The results of the comparisons were as follows: The mice in red group survived longer than the mice in green group. The survival rate of mice in the blue group was similar to the mice in the green group. **(C)** Log parasitemia data for each mice group (C57BL/6 mice infected and/or treated with anti-LFA-1) are represented as mean ± SD for each group (*n* = 4). Values from peak parasitemia (day 9) were no significantly different across the groups (*P* > 0.05). On day 14, significant difference was found between the infected and treated with anti-LFA-1 mice and infected and treated with the anti-2A3 isotype control mice (*P* < 0.05). **(D)** Kaplan–Meier curves for survival of the different groups were compared using the log-rank test. The results of the comparisons were as follows: the mice in yellow group survived longer than the mice in blue group. **(E)** Log parasitemia data for each mice group (ICAM-deficient and C57BL/6 mice) are represented as mean ± SD for each group (*n* = 4). Peak parasitemia values (day 9) were significantly different across C57BL/6 groups and in ICAM-deficient mice groups (*P* > 0.05). **(F)** Kaplan–Meier curves for survival of the different groups were compared, and there were no differences across groups. **(G)** MFI of CD54 (ICAM-1) on CD8^+^ T cells from C57BL/6 and ICAM-deficient mice: the red line indicates MFI of naïve C57BL/6 mice, whereas the blue line indicates MFI of naïve ICAM-deficient mice. One out of two individual experiments is shown.

Previously, we demonstrated that treatment with FTY720, which retains CD8^+^ T cells in the lymph nodes *via* blockade of receptor S1Pr1, culminates in death of immunized mice. As LFA-1 and VLA-4 integrins were expressed on those specific CD8^+^ T cells we investigated the role of these molecules following immunization and *T. cruzi* infection. To this end, immunized and infected mice were treated with 250 µg of monoclonal antibodies anti-LFA-1 and/or anti-VLA-4 every 48 h to block those molecules. Initially, we analyzed blood parasitemia and, as shown in Figure 1A, mice treated with anti-LFA-1 (green) antibody had increased blood parasitemia when compared with the group only immunized and treated with the control isotype (red), whereas mice treated with anti-VLA-4 (yellow) had a parasite burden similar to the immunized (red). To examine whether these two integrins exhibit synergism, one group was treated with both antibodies simultaneously (Figure 1A, blue group). Simultaneous treatment resulted in increased blood parasitemia, but this increase was not significant when compared with the group treated with anti-LFA-1 only, indicating that LFA-1, but not VLA-4, is important to control blood parasites. With respect to survival (Figure 1B), all mice treated with anti-LFA-1 died after 26 days, whereas all anti-VLA-4-treated mice and isotype control treated mice survived. Therefore, no statistical differences were observed in the survival rate between the mice treated with anti-LFA-1 (green) and the mice treated with both antibodies (blue), but there were differences in survival rate between the mice treated with anti-LFA-1 and mice treated with isotype control. Therefore, during LFA-1 blockade, mice displayed increased blood parasitemia and succumbed after challenge with *T. cruzi*, while VLA-4 blockade does not interfere with parasitemia and survival of treated mice.

To confirm the role of LFA-1 during *T. cruzi* infection, C57BL/6 mice naturally resistant to *T. cruzi* infection were infected and treated. C57BL/6 mice treatment with anti-LFA-1 was able to control blood parasitemia until 12th day after infection, but after that, the blood parasitemia increased and all mice treated with anti-LFA-1 rapidly succumbed to infection (Figures 1C,D) when compare the mice treated with the isotype control.

Since LFA-1 blockade increased mouse susceptibility to infection by *T. cruzi*, we investigated the importance of the ICAM-1 integrin, a major ligand of LFA-1. To this end, genetically ICAM-1-deficient mouse was used. These mice were immunized and infected for parasitemia and survival analysis. Both C57BL/6 and ICAM-1 knockout mice displayed similar parasitemia, and the two groups immunized with the ASP-2 gene showed a decreased parasitemia when compared with the vector control immunized groups (Figure 1E). In addition, all mice survived the experimental challenge with *T. cruzi* (Figure 1F). Figure 1G shows the expression of CD54 (ICAM-1) on spleen of CD8^+^ T cells of WT and deficient mice, and, as expected, the latter ones have lower expression of CD54 compared with WT mice. Altogether, these results indicate that the absence of ICAM-1 does not increase susceptibility to *T. cruzi* infection and suggest that, even though ICAM-1 is a major ligand of LFA-1, there is another ligand (i.e., ICAM-2) that binds to LFA-1 allowing it to exert its functions.

### LFA-1 Blockade Increases Tissue Parasite of Immunized and Infected A/Sn Mice

**Figure 2:**
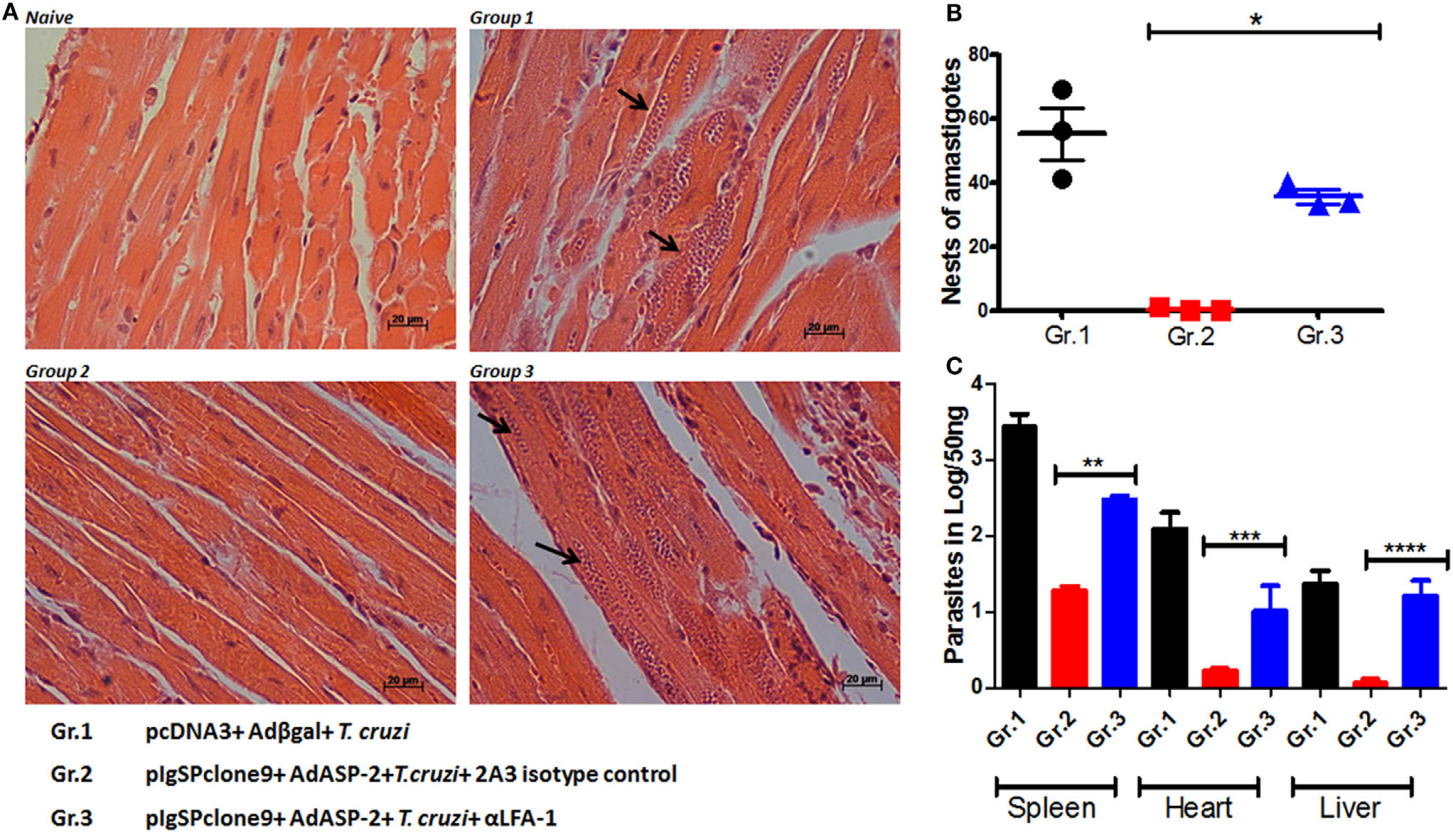
Treatment with anti-LFA-1 increases tissue parasitemia. A/Sn mice were immunized with ASP-2 using the heterologous “prime-boost” vaccination regimen, infected with 150 trypomastigotes forms of *Trypanosoma cruzi* and treated with anti-LFA-1 or isotype control until the 20th after infection. In this day, spleens, hearts, and livers of this mice were taken for quantification of the parasite DNA and count of amastigote nests in the heart. **(A)** Histological section of the heart; the arrows indicate the amastigote nests in each group. **(B)** Quantification of heart amastigote nests performed from 50 fields/group. **(C)** Quantification of the parasite DNA by real-time PCR in the different groups (Log) obtained from 50 ng of tissue DNA. Results represent mean ± SD for each group (*n* = 4) and to a single experiment. Asterisks represent statistical differences among the groups. Statistical analysis was performed using one-way ANOVA (*P < 0.001, **P = 0.000331, ***P = 0.006602, and ****P < 0.01).

Since anti-VLA-4 treatment did not interfere in the mice parasitemia and/or survival, all following experiments were performed by blocking LFA-1 integrin only. As the LFA-1 blockade leads to increased blood parasitemia and rapid death of the mice, we investigated whether the parasitic increase also occurs in the tissues of infected, immunized, and/or anti-LFA-1-treated A/Sn mice. The heart, liver, and spleen of these mice were extracted after the 20th day of infection for quantification of the parasite’s DNA by real-time PCR; in addition, the number of amastigote nests in the heart was quantified using hematoxylin–eosin staining. There was a statistical increase in the number of amastigote nests in the hearts of the LFA-1-treated mice compared with the immunized and infected group, and the largest amount of nests was found in the hearts of mice solely infected (Figures 2A,B). In addition, LFA-1 blockade resulted in the increase of parasites in the tissues analyzed compared with immunized, infected mice. The spleen showed higher parasite increase, followed by hearts and livers respectively (Figure 2C). These results demonstrate that treatment with anti-LFA-1 increases blood parasitemia, which will reflect on increased tissue parasite burden.

### LFA-1 Blockade Increases the Expression Level of the Fas/CD95 Molecule on the Surface of Effector CD8^+^ T Cells

**Figure 3:**
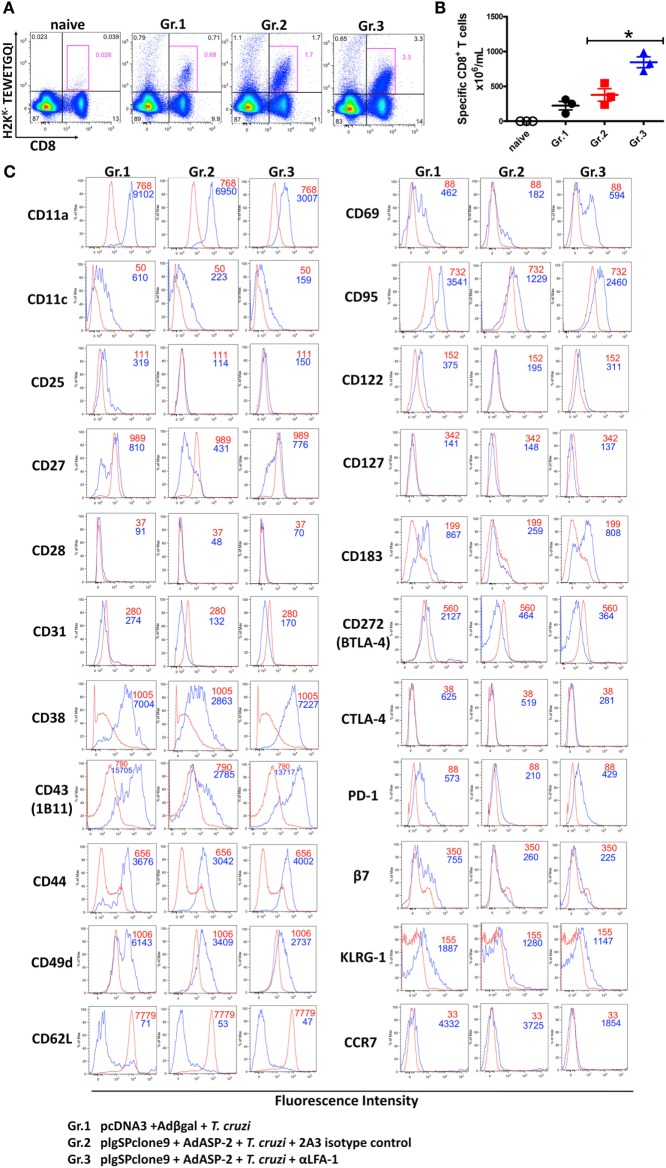
Effector CD8^+^ T lymphocytes express high levels of CD95 on the surface after anti-LFA-1 treatment. A/Sn mice were immunized with ASP-2 using the heterologous “prime-boost” vaccination regimen, infected with 150 trypomastigotes forms of *Trypanosoma cruzi* and treated with anti-LFA-1 or isotype control until the 20th after infection. In this day, the splenic cells were labeled with H2K^K^-TEWETGQI multimer, anti-CD8 and surface markers. **(A)** The frequency of specific CD8^+^ T cells in the spleen. **(B)** Absolute number of specific CD8^+^ T cells in the spleen. **(C)** Histograms with MFI of each marker analyzed in different groups. The red line represents the *naive* group, whereas the blue line represents groups 1, 2, and 3. Results in panels **(A,B)** are individual values with the mean ± SD of groups (*n* = 4), while in panel **(C)** representative analyses are shown for four mice per experiment. The experiment was performed two or more times with similar results. Statistical analysis was performed using the one-way ANOVA. Asterisks denote statistically significant differences between the groups 2 and 3 (*P* < 0.05).

We analyzed whether LFA-1 blockade affects effector phenotype and activation of specific CD8^+^ T cells. To this end, splenocytes were stained with anti-CD8 and H2K^K^ TEWETQGI-multimer, and surface markers. In previous results obtained by our group, we demonstrated that immunization followed by infection induces specific CD8^+^ T cells with the phenotype of effector cells (TE), which is characterized by the expression of CD11a^high^, CD44^high^, CD62L^low^, and CD127^low^ (25, 26). Anti-LFA-1 treatment increased the frequency and the absolute number of specific CD8^+^ T cells in the spleen (Figures 3A,B). In addition, as there is competition between the antibodies used for *in vivo* blockade and flow cytometric labeling, we observed a decrease in CD11a MFI in the anti-LFA-1-treated groups (Figure 3C). In general, the markers whose expression levels increased in the anti-LFA-1-treated group (Gr.3) in comparison with the infected group (Gr.1) or the immunized and infected group (Gr.2) were as follows: CD27, CD43, CD69, and CD95 (Figure 3C). Our group has also demonstrated that the cells generated by immunization followed by infection expressed lower levels of CD95 on the surface compared with infected only group, and these cells were also resistant to death induced by anti-CD95 (12). Moreover, markers CD183, CD38, and PD-1 also displayed increased expression levels on the surface of specific CD8^+^ T cells after treatment with anti-LFA-1 (Gr.3) compared with Gr.2 (Figure 3C). KLRG1, however, had similar MFI among the three groups, and these groups showed low expression of markers CD122, BTLA, CTLA-4, and CD25. These results suggest that anti-LFA-1 treatment does not impair specific CD8^+^ T cells in the spleen. Instead, there is a greater frequency and absolute number of these cells. In addition, the treatment did not alter the phenotype of effector CD8^+^ T cells (TE); however, we found in the spleen of treated mice that those CD8^+^ T cells expressed high levels of CD95.

### Specific CD8^+^ T Cells Accumulate in Secondary Lymphoid Organs and Decrease Migration into the Heart after LFA-1 Blockade

**Figure 4:**
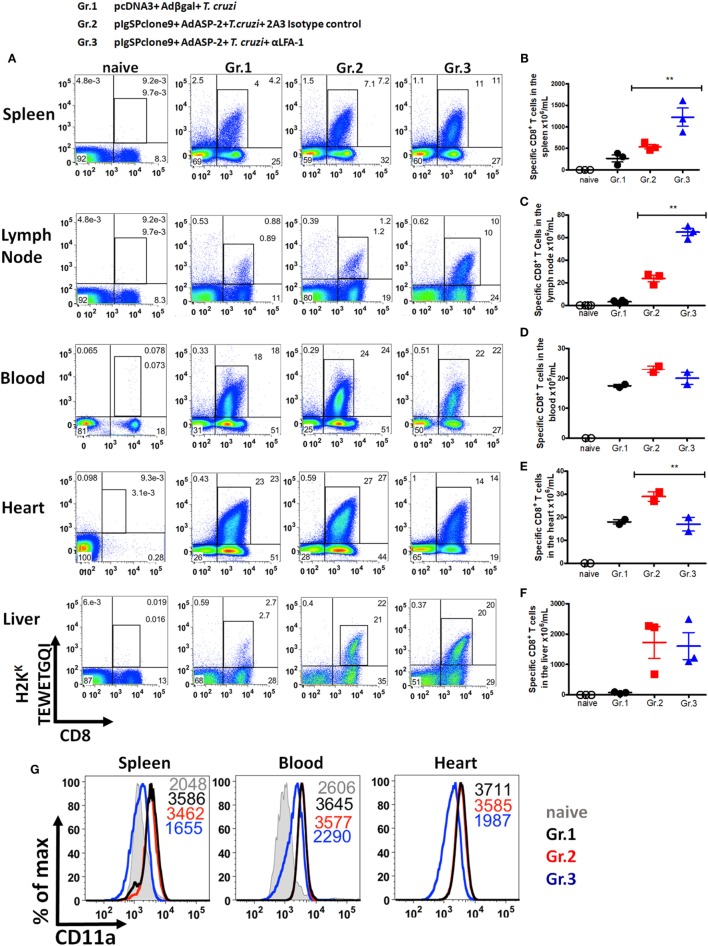
Specific CD8^+^ T cells accumulate in the spleen and lymph node and do not migrate into the heart after anti-LFA-1 treatment. A/Sn mice were immunized with ASP-2 using the heterologous “prime-boost” vaccination regimen, infected with 150 trypomastigotes forms of *Trypanosoma cruzi* and treated with anti-LFA-1 or isotype control until the 20th after infection. In this day, spleen, heart, liver, lymph nodes, and blood cells of the immunized, infected, and treated or not with anti-LFA-1 were labeled with anti-CD8 and H2K^K^-TEWETGQI multimer. **(A)** Frequency of specific CD8^+^ T cells in the spleen, lymph node, blood, heart, and liver, respectively. **(B–F)** Absolute number of specific CD8^+^ T cells in the spleen, lymph node, blood, heart, and liver, respectively. The results for the spleen, lymph node, and liver are representative values of an individual in each group (*n* = 4) with mean ± SD. While the results for the blood and heart were taken from a pool of five individuals per group, values of an individual of each repetition (*n* = 2) with mean ± SD. Statistical analysis was performed using the one-way ANOVA. **(G)** Histograms represent MFI of specific CD8^+^ T cells that express CD11a onto the surface in the spleen, blood, and heart, respectively, and the group *naïve* the MFI of CD11a was analyzed onto the surface of CD8^+^ T cells. Asterisks denote statistically significant differences between of groups 2 and 3 (**P < 0.001).

After demonstrating that anti-LFA-1 treatment increases blood and tissue parasitemia and leads to mice death, our hypothesis was that specific CD8^+^ T cells cannot migrate into the infected tissues since LFA-1 is associated with leukocyte migration. To test our hypothesis, we measured in the spleen, lymph, blood, liver, and heart the frequency of specific CD8^+^ T cells. For that propose, the cells were labeled with anti-CD8 and H2K^K^-TEWETGQI multimer. We found higher frequency and increment in the absolute numbers of specific CD8^+^ T cells in spleens and lymph nodes, but not in the blood and liver of the anti-LFA-1-treated group when compared with the infected and immunized group (Figures 4A–F). A dramatic influx reduction of specific CD8^+^ T cells after anti-LFA-1 treatment (Figure 4A) was observed in group 3 hearts. In addition, we found that specific CD8^+^ T cells in the spleen, blood, and heart of the infected group (Gr.1) and the immunized and infected group (Gr.2) expressed high levels of CD11a, whereas the cells in the immunized, infected, and anti-LFA-1-treated group (Gr.3) showed decreased CD11a MFI due to the competition described earlier (Figure 4G). We also evaluated the frequency of total CD8^+^ T cells in the spleen, blood, and heart and, as shown in Figure S2 in Supplementary Material, there was a reduction in the frequency of CD8^+^ T cells in the heart of the anti-LFA-1-treated group (Figures S2A–D in Supplementary Material). Furthermore, by immunohistochemistry, there is a decrease in the number of CD8^+^ T cells in the heart of animals treated with anti-LFA-1 (Figures S2E,F in Supplementary Material). The number of CD8^+^ T cells in the heart of the treated group was similar to the infected group, and in relation to the immunized group, treatment with anti-LFA-1 decreased the migration of CD8^+^ T cells to the heart, observed by the low number of these cells in the cardiac tissue. These results corroborate the decrease in specific CD8^+^ T cells in those organs. Therefore, during LFA-1 blockade, specific CD8^+^ T cells accumulate in the secondary lymphoid organs, such as spleen and lymph node, and cannot migrate into the heart, as observed by the lower frequency of these cells in that organ.

### Specific CD8^+^ T Cells Degranulate and Produce IFN-γ and TNF-α after Anti-LFA-1 Treatment

**Figure 5:**
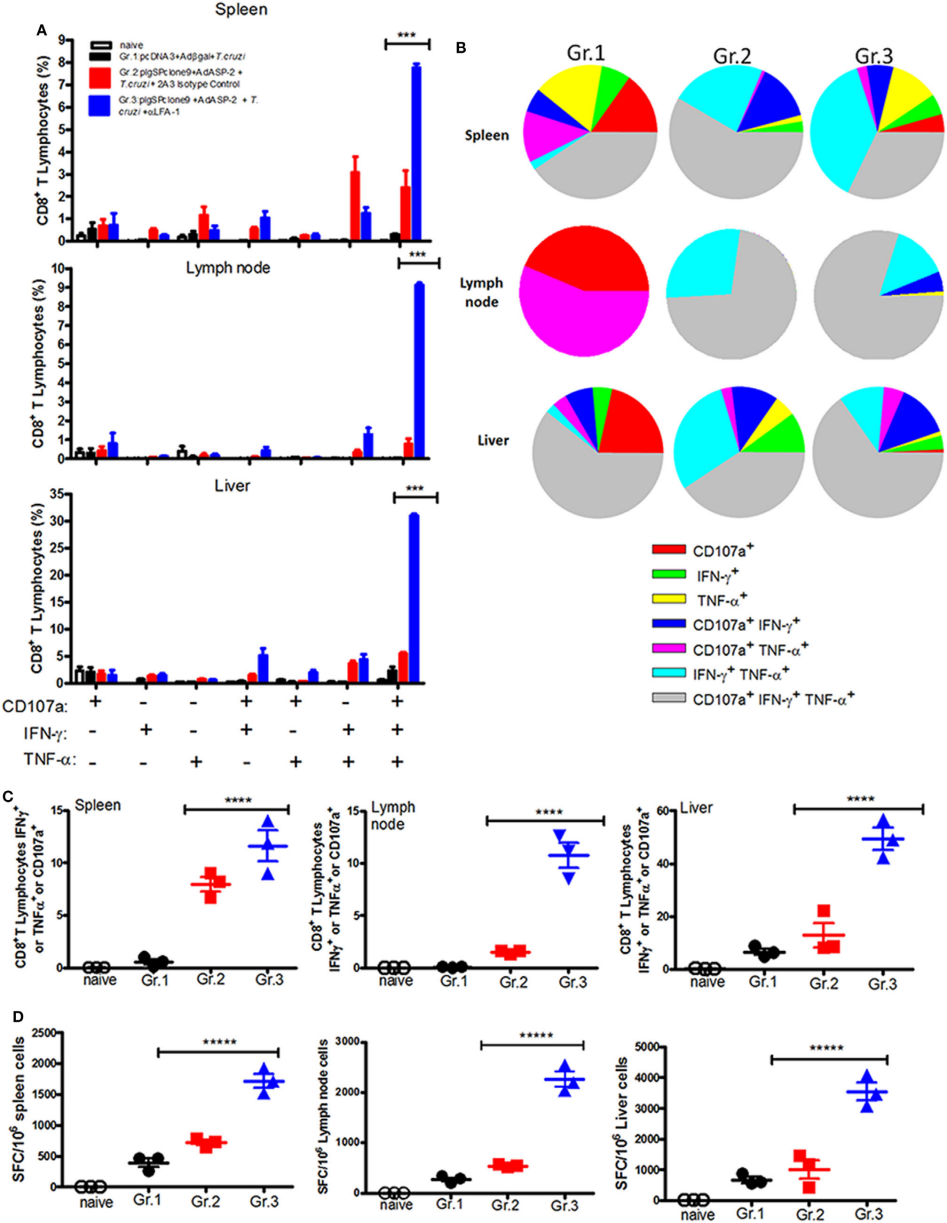
Anti-LFA-1 treatment increases the polyfunctionality of specific CD8^+^ T cells in the spleen, liver, and lymph node, and the number of IFN-γ-producing cells. A/Sn mice were immunized with ASP-2 using the heterologous “prime-boost” vaccination regimen, infected with 150 trypomastigotes forms of *Trypanosoma cruzi*, and treated with anti-LFA-1 or isotype control until 20th days after infection. In this day, splenocytes and cells of inguinal lymph nodes were collected. Furthermore, leukocytes from the liver were isolated by Percoll. These cells were restimulated *in vitro* in the presence of the peptide TEWETGQI at a final concentration of 10 mM. After 12 h, cells were stained for CD8, IFN-γ, and TNF-α. Frequencies were initially estimated for any CD8^+^ that expressed surface CD107a, IFN-γ, or TNF-α after stimulation *in vitro* with peptide TEWETGQI. **(A)** Percentage of specific CD8^+^ T cells performing each of the functions shown in the graph combinations; (+) indicates presence, while (−) indicates absence of CD107a/IFN-γ/TNF-α. **(B)** Pie chart represents the fraction of specific CD8^+^ T cells that carry each of the combinations shown in the legend. **(C)** Amplitude of the immune response, i.e., the percentage of CD8^+^ T cells that are performing at least one of the functions indicated. **(D)** ELISPOT graph of the IFN-γ-producing cells. Results are representative of two independent experiments with the mean ± SD of each individual shown in the graphs (*n* = 4). Asterisks show statistical difference between the groups. Statistical analysis was performed using one-way ANOVA (*P* < 0.05). Boolean analysis was performed using FlowJo software. SFC, spot-forming cell.

Having in mind the important role of IFN-γ during infection by *T. cruzi* (2), we analyzed whether the anti-LFA-1 treatment may alter production of IFN-γ by specific CD8^+^ T cells. We also analyzed the effector function of specific CD8^+^ T cells in the spleen, liver, and lymph nodes regarding TNF-α production and indirect cytotoxicity involving cell surface mobilization of CD107a. The gate strategy used to evaluate the production of cytokines and the polyfunctionality of specific CD8^+^ T cells is illustrated in Figures S3A,B in Supplementary Material. In the spleen of the anti-LFA-1-treated group, compared with the immunized and infected group, there was an increase in the percentage of polyfunctional specific CD8^+^ T cells, that is, cells that are capable of degranulating and, at the same time, producing IFN-γ and TNF-α. Such increase in polyfunctionality of specific CD8^+^ T cells after anti-LFA-1 treatment was also observed in lymph nodes and liver (Figures 5A,B). In addition, anti-LFA-1 treatment also culminated in an increase in the amplitude of the immune response, i.e., the percentage of specific CD8^+^ T cells producing IFN-γ or TNF-α or degranulating, in the spleen, lymph node, and liver (Figure 5C). The number of specific CD8^+^ T cells producing IFN-γ was higher in the anti-LFA-1-treated group (Gr.3), when compared with Gr.2, and this increase occurred in the spleen, the lymph node, and liver (Figure 5D). Altogether, these results show that LFA-1 blockade does not affect the effector function of specific CD8^+^ T cells regarding IFN-γ and TNF-α secretion and degranulation. The increase in the effector function is probably due to the accumulation of specific cells in spleen and lymph node after treatment with anti-LFA-1.

### Specific CD8^+^ T Cells Reduce Direct Cytotoxicity against Target Cells after Treatment with Anti-LFA-1

**Figure 6:**
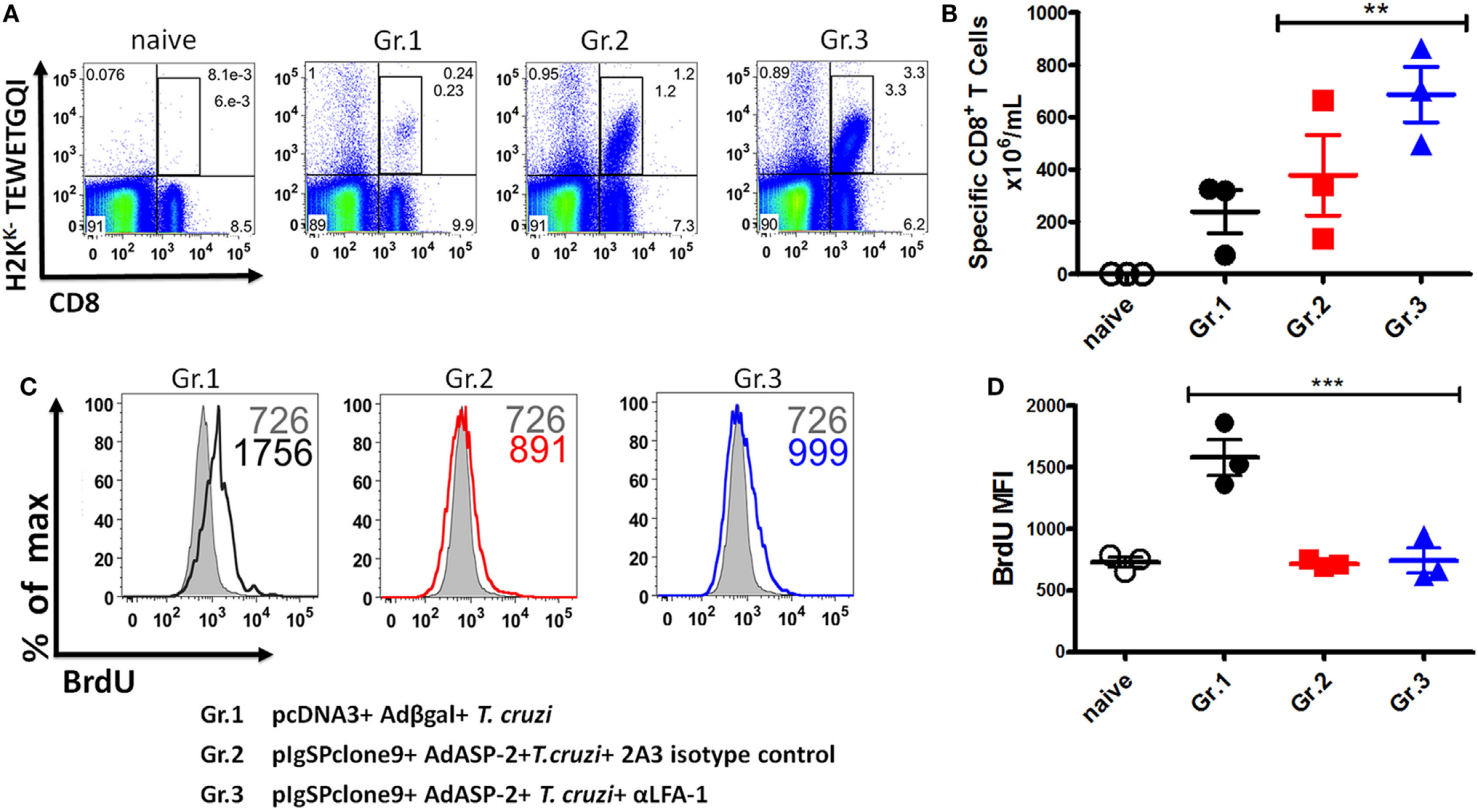
Specific CD8^+^ T cells can proliferate after the treatment with anti-LFA-1. A/Sn mice were immunized with ASP-2 using the heterologous “prime-boost” vaccination regimen, infected with 150 trypomastigotes forms of *Trypanosoma cruzi*, and treated with anti-LFA-1 or isotype control until 20th days after infection. Mice received 2 mg of 5-bromo-2′-deoxyuridine (BrdU) every 48 h. In this day, spleen was harvested for the proliferation assay. 2 × 10^6^ splenocytes were labeled with H2K^K^-TEWETGQI multimer, anti-CD8 and anti-BrdU. **(A,B)** The frequency and absolute number of specific CD8^+^ T cells in the spleen, respectively. **(C,D)** Histograms with MFI of BrdU in the groups and graph of the MFI mean of the specific CD8 T cells expressing BrdU. The graphs represent the mean ± SD for each individual (*n* = 3). Representative results of two independent experiments. Statistical analysis was performed using one-way ANOVA test (**P < 0.01; ***P < 0.005).

First, we tested whether treatment had impaired the frequency and absolute numbers of specific CD8^+^ T cells. As we can see, there was an increase in the frequency and absolute numbers of specific CD8^+^ T cells in the anti-LFA-1-treated group (Figures 6A,B). We concluded that the treatment did not interfere with specific CD8^+^ T cell expansion. Since specific CD8^+^ T cells are capable of secreting IFN-γ and TNF-α after LFA-1 blockade in the spleen, we assessed whether the treatment had impaired specific CD8^+^ T cells proliferative capacity. The proliferation of specific CD8^+^ T cells in the spleen was analyzed *in vivo* by thymidine BrdU analog incorporation. We found that a similar proportion of the H2K^K^-TEWETGQI CD8^+^ cells incorporated BrdU *in vivo* in non-treated or treated mice indicating that the proliferative capacity of these cells was not significantly different (Gr. 2 and Gr. 3, Figure 6C). However, the infected mice have a greater proliferation in comparison with groups 2 and 3 (Figures 6C,D).

**Figure 7:**
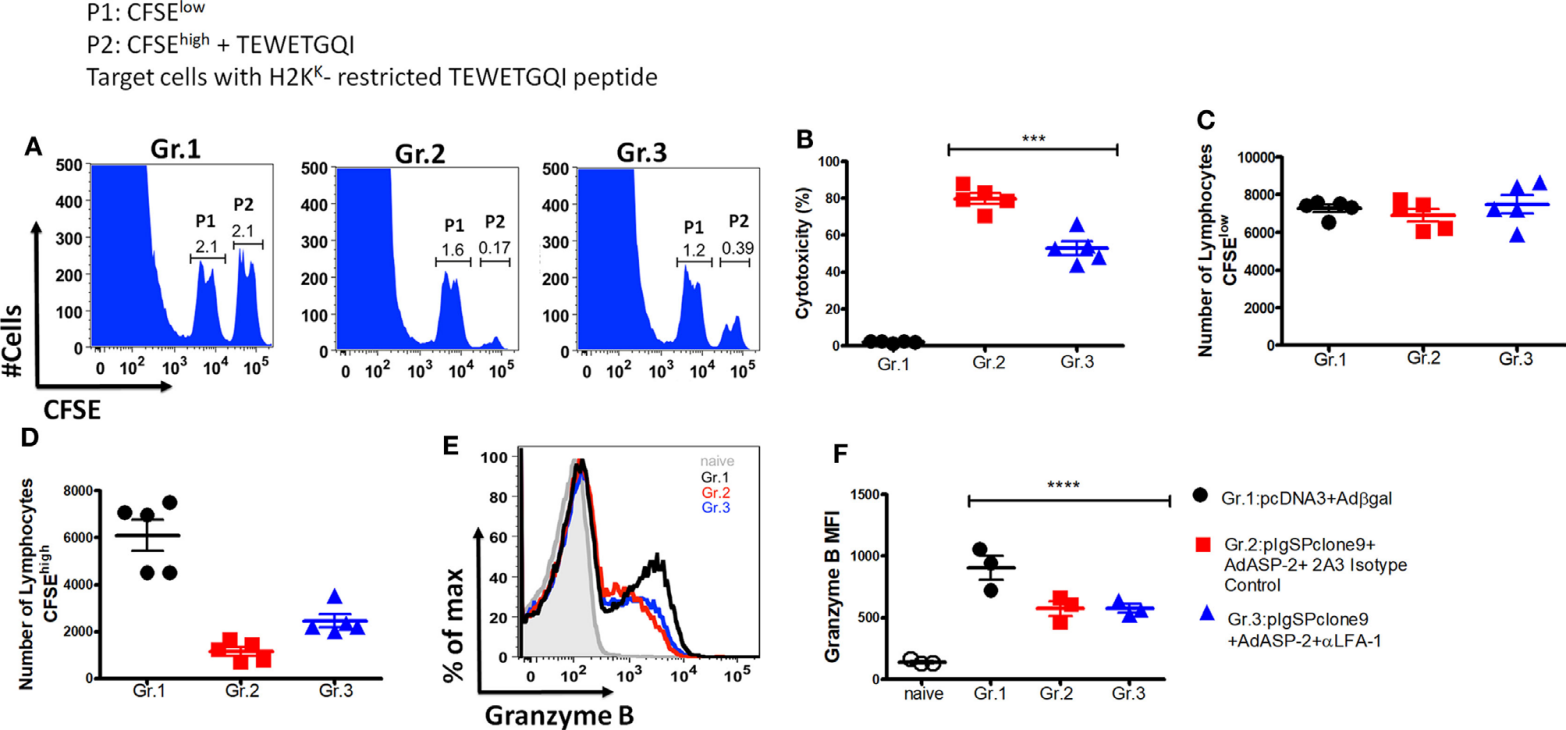
Anti-LFA-1 treatment affects the *in vivo* cytotoxicity of specific CD8^+^ T cells. A/Sn mice were immunized with ASP-2 using the heterologous “prime-boost” vaccination regimen, infected with 150 trypomastigotes forms of *Trypanosoma cruzi*, and treated with anti-LFA-1 or isotype control until 20th days after infection. After that, the spleen was harvested for the cytotoxicity assay. Splenocytes from *naive* mice were used for labeling with CFSE^low^ (1 µM) and CFSE^high^ (10 µM); the CFSE^high^ population was pulsed with the 2.5 µM of peptide TEWETGQI. The mice immunized and anti-LFA-1 treated or untreated received 10 × 10^6^ cells from each population *via* retro-orbital route. In addition, for analysis of granzyme B expression by the specific CD8^+^ T cells, 2 × 10^6^ splenocytes were stimulated *in vitro* with 10 µM TEWETGQI peptide. After 12 h, the splenocytes were collected for intracellular labeling of granzyme B. **(A)** The histograms represent the evens of CFSE^low^ (P1) and CFSE^high^ (P2) cells in each group. **(B)** Percentage of the cytotoxicity of specific CD8^+^ lymphocytes with the mean ± SD for each individual (*n* = 6). **(C,D)** The graphs with the mean cells CFSE^low^ and CFSE^high^ in the spleen of mice. **(E,F)** Histograms and the graph represent MFI and the percentage of specific CD8^+^ T cells that express granzyme B, respectively. The graphs represent the mean ± SD for each individual (*n* = 3). Representative results of two independent experiments. Statistical analysis was performed using one-way ANOVA test (***P < 0.01; ****P = 0.0047).

Another effector function triggered by specific CD8^+^ T cells is the direct cytotoxicity against the target cells. Here, we analyzed whether this function had been affected by the treatment. For that purpose, we used *in vivo* cytotoxicity assay. Figure 7A shows representative histograms containing two populations P1 (CFSE^low^) and P2 (CFSE^high^) showing the specific lysis of H2K^K^-TEWETGQI peptide-labeled CFSE^high^ cells from Gr.1, Gr.2, and Gr.3 groups (Figure 7A). Surprisingly, we observed that the immunized mice treated with anti-LFA-1 had significantly decreased cytotoxicity when compared with the immunized group (Figure 7B). The specific CD8^+^ T cells from the immunized mice have 80% cytotoxicity whereas the anti-LFA-1-treated cells showed a 29% percentage drop. Figure 7C shows that the numbers of CFSE^low^ cells were similar across groups 1, 2, and 3, while the number of cells CFSE^high^ decreased in groups 2 (non-treated) and 3 (treated) compared with group 1 (Figure 7D).

The reduction of cytotoxicity did not affect the amount of granzyme B produced by specific CD8^+^ T cells in the anti-LFA-1-treated group. We observed that MFI and the percentage of specific CD8^+^ T cells that produce granzyme B were similar across the groups (Figures 7E,F). These results demonstrate that treatment with LFA-1 directly affects the cytotoxicity of specific CD8^+^ T cells and the impairment of this function may be one of the factors responsible for the reversal protection generated by immunization observed after LFA-1 blockade.

## Discussion

Our group previously demonstrated that the recirculation of specific CD8^+^ T cells generated by heterologous prime-boost immunization and *T. cruzi* infection is of paramount importance to the protection of A/Sn mice, which are highly susceptible to infection by *T. cruzi* (12, 13). Given that integrins play an important role in cell–cell and cell–extracellular matrix interactions, and that these interactions are responsible for the intracellular signal transduction that culminates in cell migration (14) and formation of the immunological synapse (15), the aim of this study was to analyze the role of LFA-1 and VLA-4 integrins in specific CD8^+^ T cells migration. During activation of specific CD8^+^ T cells, there was an increase in the expression level of CD11a (LFA-1) and CD49d (VLA-4) chains (12). In addition, an increase in effector CD8^+^ T cells that express molecules LFA-1/ICAM-1 and VLA-4/VCAM-1 occurred in the hearts of mice infected with *T. cruzi*, suggesting the role of these molecules in the migration of specific CD8^+^ T cells into infected tissues (22, 23, 32). Here, we investigated the hypothesis that these molecules participate in the migration of specific CD8^+^ T cells generated by immunization and infection. LFA-1 blockade, but not VLA-4, makes vaccinated A/Sn and infected C57BL/6 mice susceptible to infection with *T. cruzi*. The increased susceptibility of A/Sn mice was accompanied by increased parasitemia and tissue parasite burden, as well as rapid death of these mice. Even though VLA-4 does not play any role in our model, it participates in the migration of specific CTLs into the heart of C3H/He mice when infected with the Colombian strain of *T. cruzi* (22). Because we did not see any role of VLA-4 in the mice parasitemia and survival our study was conducted toward LFA-1 role.

To analyze the role of ICAM-1, a major ligand for LFA-1, we used ICAM-1 KO mice and challenged them with blood trypomastigotes of the Y strain of *T. cruzi*, which did not affect the susceptibility of these mice to infection. This may be related to the redundancy of ligands by which a receptor can connect to alternate ligands without complete loss of functions performed by a receptor (33–35).

LFA-1 acts as a co-stimulatory molecule participating in the activation of T lymphocytes (36), and it has been shown that this molecule activates CD4^+^ T cells and induces secretion of cytokines such as IFN-γ and IL-17 (37). Therefore, we evaluated whether LFA-1 blockade impairs the activation of specific CD8^+^ T cells or interferes with phenotype of effector CD8^+^ T cells (TE). Our group has characterized the profile of CD8^+^ T cells induced by immunization that displays a phenotype of effector CD8^+^ T cells (TE). TE cells are characterized by expression of CD44^high^, CD62L^low^, and CD127^low^ (26), and we evaluated this profile and other activation markers on the cells from anti-LFA-1-treated mice. The anti-LFA-1 blockade decreased the CD11a MFI on specific CD8^+^ T cells surface by approximately 50%. In addition, treatment did not affect the phenotype of effector CD8^+^ T cells as well as the expression of early and late activation markers, such as CD69 and CD44, respectively. Results obtained by Gérard and colleagues also showed that the absence of LFA-1 does not reduce the expression of CD69 on CD8^+^ T cells (38).

The lower expression of CD95 in the cells induced upon vaccination was the main difference to CD8^+^ T cells generated by the infection, which had higher levels of CD95 (25). There is an increase in the expression of Fas/CD95 on some specific CD8^+^ T cells after immunization and treatment with anti-LFA-1. This result suggests that specific CD8^+^ T cells in mice treated with anti-LFA-1 may be more susceptible to programmed cell death by the extrinsic pathway. Similar results were shown by Borthwick and colleagues, who found that LFA-1 blockade reduces the survival of T lymphocytes, thus suggesting the important role of LFA-1 in survival signals during the process of T cells migration (39). Otherwise, reduction of migration to tissues upon LFA-1 blockade might increase effector CD8 T-cells expressing Fas in the secondary lymphoid organs.

Indeed, there is an impaired migration of specific CD8^+^ T cells after LFA-1 blockade. The number of these cells was quantified in the spleen, heart, liver, lymph nodes, and blood after anti-LFA-1 treatment. The treatment led to an increase in the frequency and absolute number of specific CD8^+^ T cells in the spleen and lymph node, and a decreased frequency mainly in the heart. However, despite the apparent decrease in the overall number of CD8 T cells in blood, this decrease was not statistically significant (Figure S2 in Supplementary Material). We have previous described that specific-peptide CD8^+^ activation occurs in the lymph nodes after subcutaneous infection by *T. cruzi* and also in vaccinated model (12, 13). We approached this subject by administering the immunosuppressive drug FTY720 (12, 13). In both models, this drug reduced lymphocyte recirculation by interfering with T cell signaling *via* S1Pr1. This interference resulted in inhibition of S1Pr1 signaling, effectively trapping T cells within the lymph node without inhibiting T cell activation. FTY720 administration significantly impaired protective immunity supporting the hypothesis that T cell recirculation is critical for the protective immunity they mediate (12, 13). Here, we have not addressed how these cells accumulate more in the lymph after treatment with anti-LFA-1. However, our data confirm that recirculation of these cells is necessary to exert their effector function in the peripheral tissues. Thus, blocking the integrin LFA-1, we observed the same accumulation not only in the lymph node but also in the spleen. However, in our immunization, infection, and treatment model, there was no change in the number of specific CD8^+^ T cells in the peripheral blood (Figure S2 in Supplementary Material), probably because of the increased accumulation of CD8^+^ T cells in the spleen and lymph node. This phenomenon may be specific to our immunization and infection model but has not yet been explored in details or found in another model. One explanation might be that the blockage of LFA-1 integrin expressed by CD8^+^ T cells prevents the interactions with its ligand might be required to exit from lymph node. Another explanation might be due the decreased speed or movement of these cells (40). These issues need to be further addressed in our vaccination and infection model. These results confirm that LFA-1 is important to the migration of specific CD8^+^ T cells into infected tissues such as the heart, and the decline of these cells should be one of the causes for increased parasitemia in that organ. Recent studies have shown that LFA-1 blockade, and not VLA-4, reduces migration speed of T lymphocytes, leading to decreased antigen scanning by T cells (41) and, hence, lower immune response and higher parasitemia.

We also evaluated whether anti-LFA-1 treatment would affect the effector function of specific CD8^+^ T cells, since these cells accumulated in the spleen are incapable of controlling the number of parasites. We analyzed the production of pro-inflammatory cytokines, crucial for controlling *T. cruzi* multiplication, such as IFN-γ and TNF-α, and observed that there was accumulation of polyfunctional specific CD8^+^ T cells capable of degranulating and simultaneously producing TNF-α and IFN-γ in the spleen, lymph node and liver. As the LFA-1 blockade retained CD8^+^ T cells in the spleen and lymph node, we believe that this accumulation led to increased effector function of these cells. In the liver, in which there was no accumulation of such cells, the increase in intracellular cytokine production can be explained by the fact that LFA-1 blockade does not affect the production of those cytokines. In addition, it has been shown that high doses of anti-LFA-1 were required for impairing the production of those mediators (42). Finally, specific CD8^+^ T cells proliferated after LFA-1 blockade, and this result was consistent with the data obtained by Gérard et al. (38).

Another important function triggered by specific CD8^+^ T cells is the direct cytotoxicity against target cells. NK cytotoxic cells and CTLs of chagasic patients express perforin and granzyme B, suggesting the importance of these mediators to the host immune response (43). In addition, specific CD8^+^ T cells induced by immunization are cytotoxic and can produce perforin (2). Anti-LFA-1 treatment decreased the 80% cytotoxicity of specific CD8^+^ T cells in the immunized group to 60% after treatment. Similar results were obtained by Petit et al., whereas LFA-1 blockade was responsible for a 50% decrease of direct cytotoxicity triggered by CD8^+^ T cells (42). In addition, the decrease in cytotoxicity is independent of cytotoxic granule production and degranulation because there was no decrease in the amount of granzyme B and CD107a in specific CD8^+^ T cells treated with anti-LFA-1. We believe that cytotoxicity is impaired not because of the reduction of cytotoxic granules but because LFA-1 is important to maintain stability between target and cytotoxic cells. The role of LFA-1 in maintaining a stable contact between the cells was demonstrated by blocking β2 chain of LFA-1, since absence of such molecule impaired the formation time of cell protrusions, as well as the stability of the immunological synapse (44–47). Also, it has been shown that LFA-1 blockade impairs close contact between effector T cells and antigen-presenting cells (48).

The reduction of direct cytotoxicity may explain why specific CD8^+^ T cells cannot control the number of parasites in the spleen even if accumulation of these cells occurs during LFA-1 blockade. In addition, our results suggest that LFA-1 plays an important role in the migration of specific CD8^+^ T cells into the heart and the survival of these cells. Finally, we believe that impairment of direct cytotoxicity and lower migration of specific CD8^+^ T cells into the heart are the major causes of lack of protection to *T. cruzi* infection upon immunization and treatment with anti-LFA-1.

## Ethics Statement

This study was carried out in strict accordance with the recommendations in the Guide for the Care and Use of Laboratory mice of the Brazilian National Council of Animal Experimentation (http://www.cobea.org.br/). The protocol was approved by the Committee on the Ethics of Animal Experiments of the Institutional Animal Care and Use Committee at the Federal University of Sao Paulo (Id # CEP 7559051115).

## Author Contributions

CF, LC, and JV conceived and designed the experiments. CF, LC, FV, BM, CM, PR, and DA performed the experiments. CF and LC analyzed the data and prepared the figures. AM, RG, O-BR, and MR contributed with reagents and materials. CF and JV wrote the manuscript. ML, JL-V, CM, and BM performed the final review of the article. All the authors read and approved the final article.

## Conflict of Interest Statement

The authors declare that the research was conducted in the absence of any commercial or financial relationships that could be construed as a potential conflict of interest.
