# Supplementary material for: LFA-1 Mediates Cytotoxicity and Tissue Migration of Specific CD8+ T Cells after Heterologous Prime-Boost Vaccination against Trypanosoma cruzi Infection
Source: Front Immunol. 2017 Oct 13;8:1291. doi: 10.3389/fimmu.2017.01291 (PMC5645645; doi:10.3389/fimmu.2017.01291)
Supplement: Supplementary file 1 [file Data_Sheet_1.PDF]

Supplementary Material

LFA-1 Mediates Cytotoxicity and Tissue Migration of Specific CD8$^+$ T Cells After Heterologous Prime-Boost Vaccination Against *Trypanosoma cruzi* Infection

Camila Pontes Ferreira[1,2#], Leonardo Moro Cariste[1,8#], Fernando Dos Santos Virgílio[1,2], Barbara Ferri Moraschi[1,2], Caroline Brandão Monteiro[8], Alexandre Vieira Machado[3], Ricardo Tostes Gazzinelli[3,4], Oscar-Bruna Romero[5], Pedro Luiz Menin Ruiz[8], Daniel Ribeiro Araki[8], Joseli Lannes-Vieira[6], Marcela de Freitas Lopes[7], Mauricio Martins Rodrigues[1,2§], José Ronnie Carvalho de Vasconcelos [1,2,8*]

[1]Molecular Immunology Laboratory; Center of Molecular and Cellular Terapy; [2]Department of Microbiology, Immunology and Parasitology; Federal University of São Paulo (UNIFESP), São Paulo, Brazil; [3]René Rachou Research Center, Fiocruz, Minas Gerais, Brazil; [4]Division of infectious Disease and Immunology, Departament of Medicine, University of Massachusetts Medical School, Worcester, Massachusetts, U.S.A; [5]Departamento de Microbiologia, Imunologia e Parasitologia, Universidade Federal de Santa Catarina, - Florianopolis, SC, Brazil; [6]Biology Interactions Laboratory, Oswaldo Cruz Institute, Fiocruz, Rio de Janeiro, Brazil; [7]Institute of Biophysics Carlos Chagas Filho, Federal University of Rio de Janeiro, Rio de Janeiro, Brazil; [8]Department of Biosciences of the Federal University of São Paulo-Baixada Santista, Brazil.

[*]Corresponding author: José Ronnie Carvalho de Vasconcelos.

[#]These authors contributed equally to this work

[§]In memoriam

e-mail: jrcvasconcelos@gmail.com.br

Supplementary Figure 1. The *in vitro* treatment with anti-LFA-1 decreases the expression of CD11a on the surface of total CD8 + T cells. To evaluate the blockade of the CD11a (LFA-1) molecule, splenocytes from naive and infected animals were incubated with 250 µg/ml of 2A3 isotype control or with anti-LFA-1. **(A)** The histograms represent the CD11a MFI on gated CD8+T cells harvested from infected mice and treated *in vitro* for 24 hours with 250 µg/mL of 2A3 isotype control or anti-LFA-1. **(B)** The histograms represent the CD11a MFI on gated CD8+T cells harvested from naive mice were stimulated with 1µg/mL of anti-CD3 and incubated for 24 hours with 250µg/mL of 2A3 isotype control or anti-LFA-1. Results are representative of two independent experiments. The MFI of CD11a was analyzed by FlowJo software.

Supplementary Figure 2. LFA-1 blockade decreases the amount of CD8+ T cells in the heart. Spleen, heart, and blood cells of immunized, infected, and ant-LFA-1-treated or untreated mice were labeled with anti-CD8 after the 20th day of infection. **(A)** Dot plots with gate in the CD8 T cells in the spleen, blood and heart. **(B-D)** The graphics with the mean of CD8+ T cells in the spleen, heart, and blood respectively. The results for blood and heart were taken from a pool of 5 individuals per group. **(E)** Immunohistochemistry staining of CD8 T cells infiltrating the cardiac tissue at 20 dpi, DAPI was used to reveal the nucleus of the cells CD8+ T cells. **(F)** Number of CD8+ T cells in 50 microscopic fields of cardiac tissue from A/Sn mice. Results are representative of two independent experiments with the mean ± SD of each individual shown in the graphs (n=2). The frequency of CD8+ T cells was obtained from the analysis of FlowJo software. Asterisks show statistical difference between the group 2 and 3. Statistical analysis was performed using One-way ANOVA (***P<0,05 and *****P<0,001).

Supplementary Figure 3. Gate strategy used to analyze the intracellular staining. A/Sn mice were immunized with ASP-2 using the heterologous 'prime-boost' vaccination regimen, infected with 150 tripomastigotes forms of *T. cruzi* and treated with anti-LFA-1 or isotype control until the 20th after infection. In this day the splenic cells were re-stimulated *in vitro* in the presence of the peptide TEWETGQI at a final concentration of 10 mM. After 12 h, cells were stained for CD8, IFN-γ, and TNF-α. **(A)** The gate strategy was made as follows: SSC-A/Time, SSC-A/FSC-A, FSC-H/FSC-A and SSC-A/CD8. **(B)** The dot-plot graphs represent the gate strategy used to analyze the production of intracellular cytokines in peptide-stimulated CD8+ T cells in the spleen of the mice.
