# Supplementary figures and images for: LFA-1 Mediates Cytotoxicity and Tissue Migration of Specific CD8+ T Cells after Heterologous Prime-Boost Vaccination against Trypanosoma cruzi Infection
Source: Front Immunol. 2017 Oct 13;8:1291. doi: 10.3389/fimmu.2017.01291 (PMC5645645; doi:10.3389/fimmu.2017.01291)

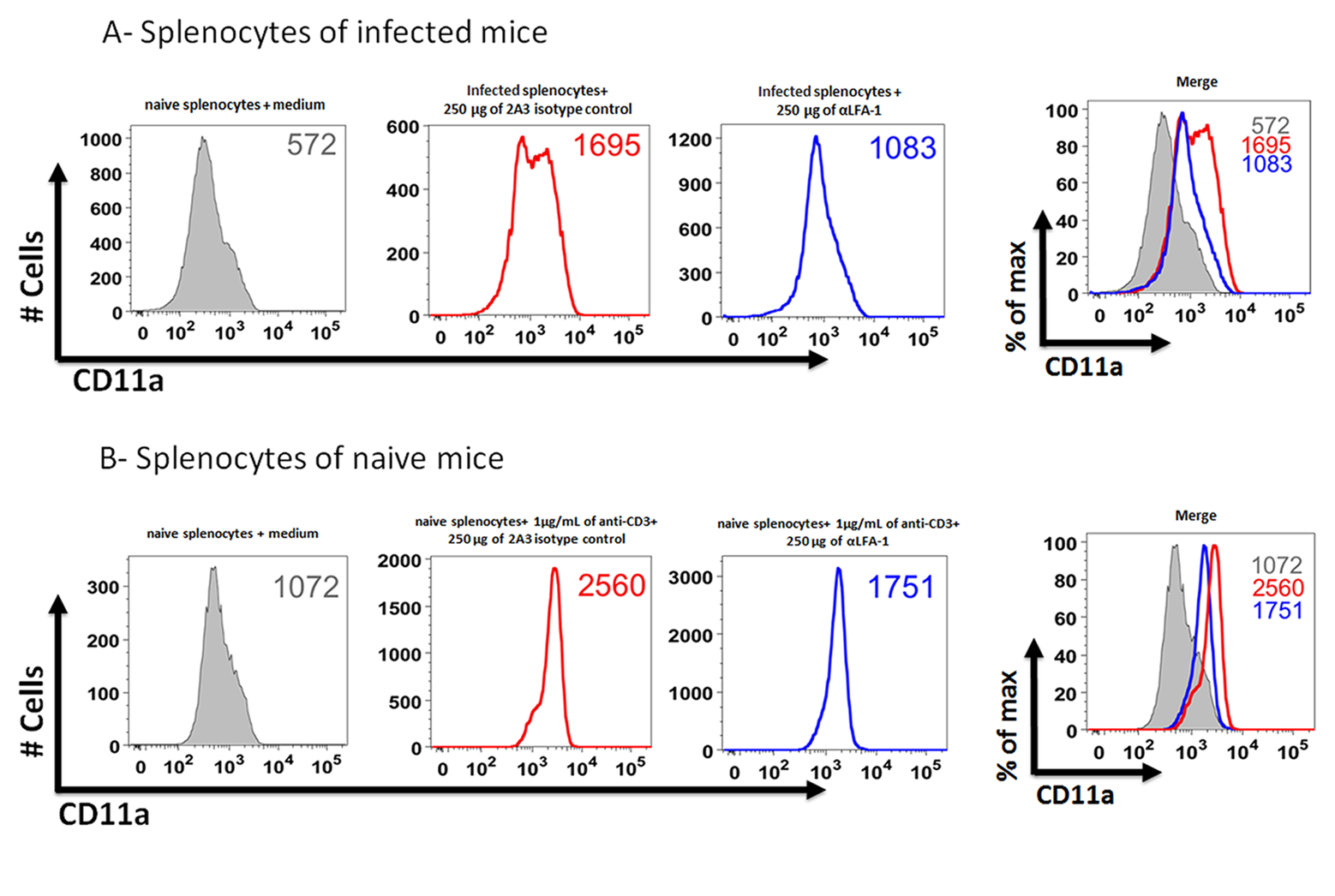

Supplement: Supplementary file 2 [file Image_8.TIF]

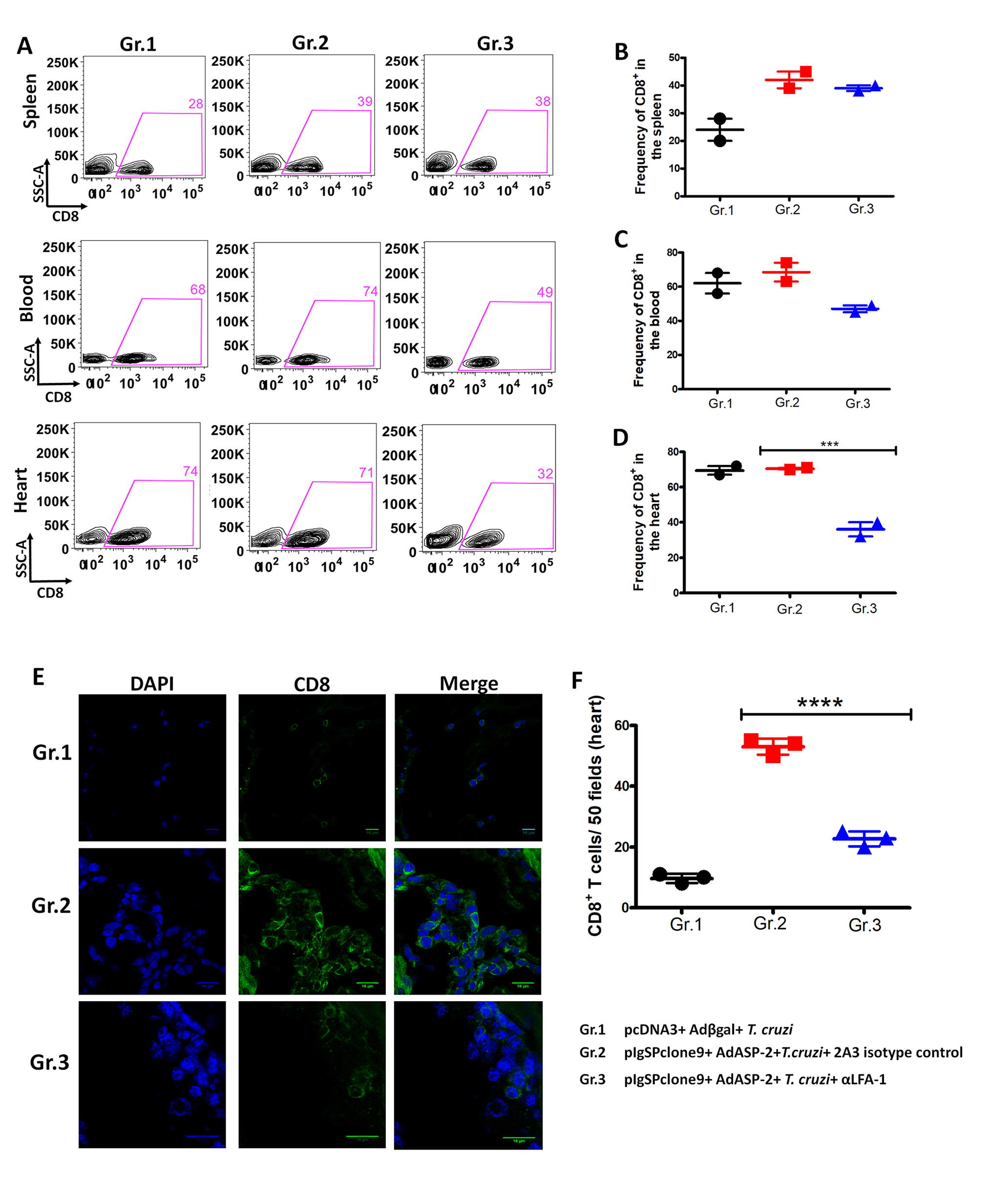

Supplement: Supplementary file 3 [file Image_9.TIF]
